# Effect of Joint Friction Compensation on a “Muscle-First” Motor-Assisted Hybrid Neuroprosthesis

**DOI:** 10.3389/fnbot.2020.588950

**Published:** 2020-12-11

**Authors:** Ryan-David Reyes, Rudolf Kobetic, Mark Nandor, Nathaniel Makowski, Musa Audu, Roger Quinn, Ronald Triolo

**Affiliations:** ^1^Advanced Platform Technology Center, Department of Veterans Affairs, Louis Stokes Cleveland Department of Veterans Affairs Medical Center, Cleveland, OH, United States; ^2^Department of Biomedical Engineering, Case Western Reserve University, Cleveland, OH, United States; ^3^Department of Mechanical Engineering, Case Western Reserve University, Cleveland, OH, United States; ^4^Department of Physical Medicine & Rehabilitation, MetroHealth Medical Center, Cleveland, OH, United States

**Keywords:** motorized, exoskeleton, metabolic, consumption, METs, friction, compensation

## Abstract

This study assessed the metabolic energy consumption of walking with the external components of a “Muscle-First” Motor Assisted Hybrid Neuroprosthesis (MAHNP), which combines implanted neuromuscular stimulation with a motorized exoskeleton. The “Muscle-First” approach prioritizes generating motion with the wearer's own muscles via electrical stimulation with the actuators assisting on an as-needed basis. The motorized exoskeleton contributes passive resistance torques at both the hip and knee joints of 6Nm and constrains motions to the sagittal plane. For the muscle contractions elicited by neural stimulation to be most effective, the motorized joints need to move freely when not actively assisting the desired motion. This study isolated the effect of the passive resistance or “friction” added at the joints by the assistive motors and transmissions on the metabolic energy consumption of walking in the device. Oxygen consumption was measured on six able-bodied subjects performing 6 min walk tests at three different speeds (0.4, 0.8, and 1.2 m/s) under two different conditions: one with the motors producing no torque to compensate for friction, and the other having the motors injecting power to overcome passive friction based on a feedforward friction model. Average oxygen consumption in the uncompensated condition across all speeds, measured in Metabolic Equivalent of Task (METs), was statistically different than the friction compensated condition. There was an average decrease of 8.8% for METs and 1.9% for heart rate across all speeds. While oxygen consumption was reduced when the brace performed friction compensation, other factors may have a greater contribution to the metabolic energy consumption when using the device. Future studies will assess the effects of gravity compensation on the muscular effort required to lift the weight of the distal segments of the exoskeleton as well as the sagittal plane constraint on walking motions in individuals with spinal cord injuries (SCI).

## 1. Introduction

Spinal cord injury affects 291,000 people in the US with 17,000 new cases per year (NSCISC, 2019). Injuries of the thoracic spinal cord result in paraplegia, which compromises muscular and/or sensory function in the trunk and lower extremities that can impair the ability to walk. Individuals with paraplegia rate restoration of walking as one of their highest priorities (Anderson, 2004). Ambulatory motion can be restored to this population via several methods.

Functional Neuromuscular Stimulation (FNS) technologies, often referred to as neuroprostheses, can restore some ambulatory motion to the affected population by electrically exciting the intact peripheral nerves below the injury to contract the otherwise paralyzed muscle groups at the appropriate time and intensity. Neural stimulation can be delivered via electrodes applied to the surface of the skin, implanted in the muscles, or cuffs wrapped around the nerves of interest. Coordinated stimulation of the muscle groups used for gait can produce motions that allow users to stand and support their body weight on their legs, or ambulate short distances with crutches, walkers, or assisted by others to maintain balance (Shimada et al., 1996; Kobetic et al., 1997; Brissot et al., 2000; Uhlir et al., 2000; Agarwal et al., 2003).

FNS can be combined with external, passive mechanical bracing, to constrain motions in the sagittal plane and couple the actions of various joints. One example of such a brace is the reciprocating gait orthosis. This class of orthoses enforce kinematic constraints to facilitate certain types of walking, such as locked-knee stiff-legged walking with the hip joints constrained to always move in opposite directions. As such, the FNS subsystem would only have to activate the hip muscles to produce this type of gait (Isakov et al., 1992).

Passive mechanical braces can be enhanced with powered actuators at the hips and knees that produce ambulatory motion for the user, or pilot. Commercial exoskeletons such as the ReWalk, Indego, and Ekso, are examples of this type of solution (Miller et al., 2016; Gad et al., 2017; Tefertiller et al., 2018). They are donned by the individual, and the exoskeleton provides all power to produce the motions of the step.

Devices such as the Motor Assisted Hybrid Neuroprosthesis (MAHNP) designed by our team, Kinesis (del Ama et al., 2014), and the SEAHO (Kirsch et al., 2014) combine the two paradigms of FNS and active exoskeletal bracing to produce a class of systems known as Hybrid Neuroprostheses or Hybrid Exoskeletons (del Ama et al., 2012; Anaya et al., 2018). These systems integrate muscles and motors in tandem in order to produce ambulatory motions in their pilots. These approaches combine the consistency and controllability of motorized exoskeletons with the rehabilitative advantages of FNS to combat muscle atrophy and reap other health benefits.

An important consideration in the design of hybrid devices is to minimize characteristics that impede the forces generated by the muscles of the pilot in order to allow the pilot to move the system under their own muscular power. Some characteristics that may impede pilot motion are the passive resistance of the motorized joints, exoskeleton weight, and movement constraints imposed by the exoskeleton structure. If the contracting muscles are impeded or constrained by these characteristics, then contractile force will be wasted and this will be reflected in an increased metabolic cost of walking using the device (Waters and Mulroy, 1999; Browning et al., 2007). The metabolic cost is a measure of how much energy an individual consumes when doing a particular activity, and this is quantified via measurements of the rate of oxygen consumption ($\overset{∙}{{\text{VO}}_{2}}$) and Metabolic Equivalent of Task (METs) (Kenny et al., 2017). Ideally, the hybrid system should be as mechanically transparent to the user as possible, such that the metabolic cost of actions such as ambulation is the same with and without the device. The metabolic cost of walking has been assessed in several hybrid and non-hybrid devices that restore ambulatory motion to the SCI population, such as the Hybrid Neuroprosthesis (Chang et al., 2017), Ekso (Kressler et al., 2018), Indego (Evans et al., 2015), ReWalk (Asselin et al., 2015), and others (Isakov et al., 1992; Israel et al., 2006; Hornby et al., 2012; Miller et al., 2016).

One of the unique features of the MAHNP developed in our lab is that the joint actuators are backdrivable, taking <6 N m of torque at all joint speeds to move the hip and knee joints when they are not locked. Because of this, SCI pilots can produce the majority of forces needed for ambulatory motions through activation of their paralyzed muscles via neural stimulation. This also results in able-bodied users being capable of walking under their own volition in the MAHNP. The purpose of this study was to quantify the effects of the passive resistance at the joints on the metabolic cost of able-bodied walking in the device at various speeds indicative of functional ambulation in the community (Robinett and Vondran, 1988; Lapointe et al., 2001).

## 2. Methods

### 2.1. Device Design

The MAHNP has four actuators, two each at the knees and hips respectively. The actuators are connected to the limbs via transmissions that provide appropriate speed reduction. The primary joint transmission at each joint is a 100:1 strain wave transmission (Harmonic Drive, Peabody, MA), connected through a spur gear set to a brushless DC outer rotor motor (Maxon Precision Motor, Switzerland). Overall gear reduction is 157:1 for the hip joints, and 100:1 for knee joints. The knee and hip actuators are capable of 8.12 and 13.19 N m of continuous torque, respectively. All four actuators are capable of 36 N m peak torque, have solenoid locking mechanisms (Thomson Linear, Redford VA), weigh 2.2 kg each, and record velocity and position via internal potentiometers. The actuators are wired to a backpack that houses the control electronics, batteries, and an FNS controller board. The FNS board interfaces with either two 12-channel percutaneous stimulator boards or one implant controller board for a 16-channel implanted stimulator-telemeter (Smith et al., 1998). However, as this study focused on able-bodied walking, the FNS capabilities of the exoskeleton were not activated.

**Figure 1:**
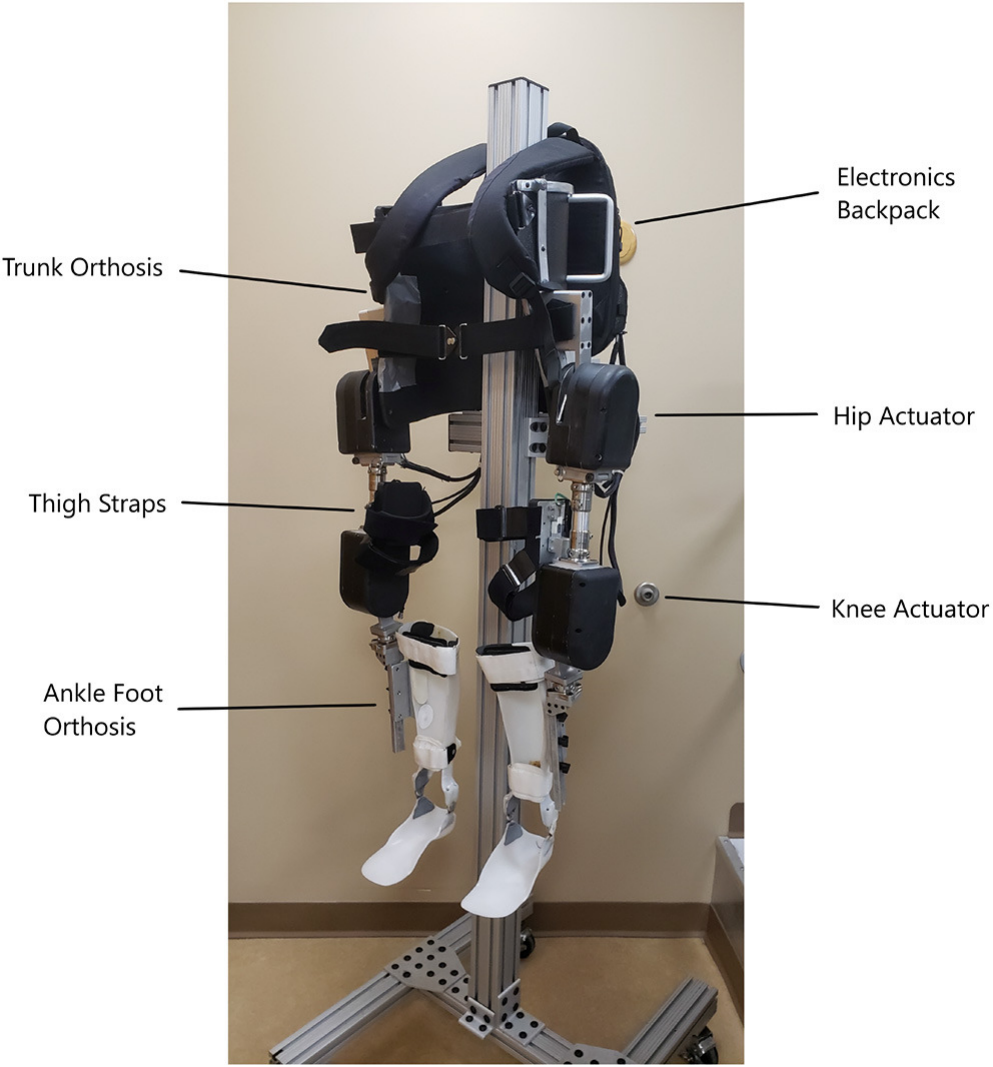
The MAHNP exoskeleton.

The hip actuators are connected to a fitted, reinforced thoracic-lumbo-sacral orthotic corset which keeps the system in place on the pilot and constrains motion of the pilot's pelvis and lower torso. Off-the-shelf adjustable pylons for prostheses are used to connect the hip actuators to the knee actuators, and allow for static angle adjustments in the sagittal and coronal planes for best fitment. The knee actuator connects to a shank piece which is attached to an ankle-foot orthosis (AFO) which holds the lower limb snug with the exoskeleton. The AFO limits motion of the shank and foot to the sagittal plane, but allows free ankle dorsiflexion and plantarflexion over the natural range of motion. Velcro straps on the trunk orthosis, thigh uprights, and ankle-foot orthoses fasten the exoskeleton system to the user and their limbs. Figure 1 depicts the MAHNP without a pilot.

### 2.2. Friction Model

**Figure 2:**
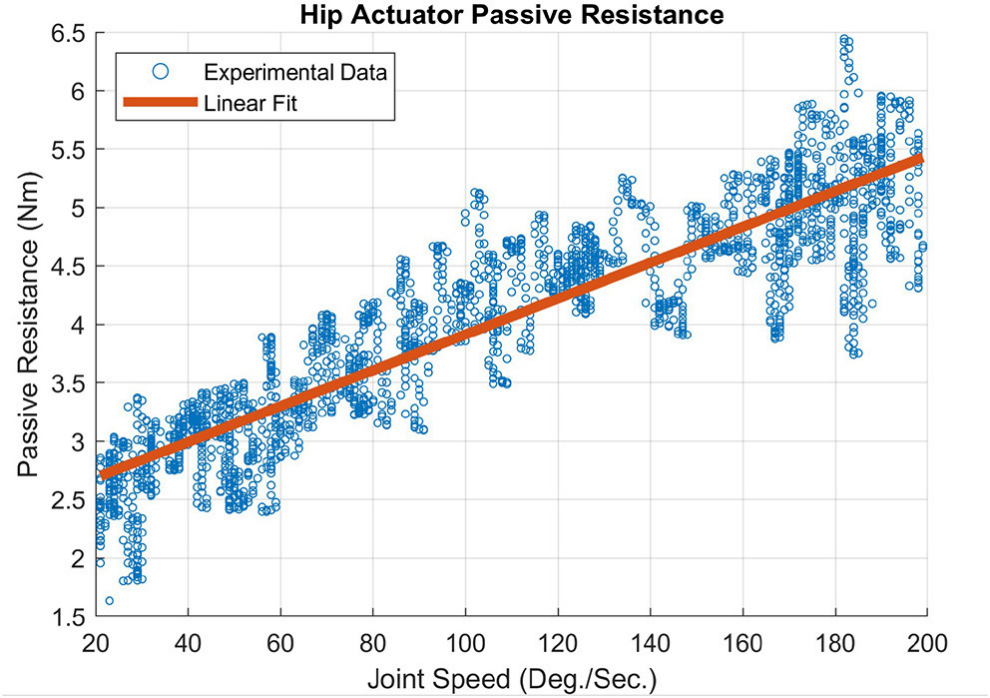
Passive resistance of the hip joint actuator.

An empirically derived passive resistance or “friction” model was developed that captures the characteristics of the viscous damping and friction in the joint actuators of the MAHNP. These values for the actuators were measured via no-load tests. The output arm that connects the actuator to the rest of the system was removed to allow the actuator to spin freely. The motor was commanded to move the output at a constant acceleration of 25 °/s^2^ from 0 °/s to 200 °/s, and the resulting current to velocity relationship was recorded. These data were integrated with isometric torques at corresponding currents to determine the relationship between joint speed and passive resistance. A linear regression was performed to fit the torque to velocity relationship, which is shown for the hip actuator in Figure 2 and resulted in the following equation to represent the inherent friction: 0.015ω + 2.38, *R*^2^ = 0.816. ω is the angular velocity of the joint, and the constant value is the maximum amount of torque commanded to the motor required before the output was able to turn, which is the static friction. The slope of the linear fit is the viscous damping constant. The knee friction characteristics were nearly identical to the hip characteristics shown here.

With these linear regressions, a sigmoidal model was developed that provides a continuous model of friction in both directions of rotation, seen in Equation (1).

(1) $$\begin{array}{r} \tau =\sigma \left(\omega \right)\cdot |\beta \cdot \omega +\gamma | \end{array}$$

τ is the computed output torque of the joint in Newton-meters. σ(ω) = 2/(1+*e*^−ω^)−1, is a shifted and scaled sigmoid function to make the linear regime centered at zero, and the range of the function from −1 to 1, which provides continuity in both directions of rotation. ω is the angular velocity of the joint, β and γ are the empirically derived constants that fit the viscous damping and static friction of the joint respectively, as seen in the regression above. Using this model, a feedforward friction compensation model was developed that allows the motor to inject power as a function of speed, in order to overcome the viscous damping. Other terms, shown in Equation (2), were added to mitigate model inaccuracy and to smooth the transition of the system when changing directions.

(2) $$\begin{array}{r} \tau =\alpha \cdot \sigma \left(\frac{\omega }{\phi }\right)\cdot |\beta \cdot \omega +\gamma | \end{array}$$

Equation (2) adds two tuning parameters to Equation (1): α and ϕ. These parameters account for the slight inaccuracy of the polynomial fits, reduce the sensitivity to noise when operating around zero degrees per second, and ensure enough torque was provided to minimize viscous damping during motion. α = 0.8 across all joints, meaning that the feedforward model compensated for 80 % of the derived viscous damping. ϕ = 4 across all joints, decreasing the slope of the linear regime of the sigmoid function to reduce sensitivity to noise between 0 and 20 degrees per second. In this low speed band, <80 % of the friction was compensated for. β and γ did not change per subject, as they are a function of the motor and transmission, and they did not change with the addition of the tuning parameters.

This study examined the difference in metabolic cost between the following two conditions. The first condition was the MAHNP without compensation, where the joints had no additional power injected to overcome internal friction. The second was with friction compensation, where the MAHNP injected power to overcome friction according to the model defined by Equation (2).

### 2.3. Study Subjects

Six able-bodied individuals (S1–S6) with no history of musculoskeletal, cardiovascular, pulmonary, or vestibular compromise volunteered for the study. Subjects had a median age of 33.5, height of 1.73 m, and weight of 70 kg. The physical characteristics of all volunteers are specified in Table 1. This study was approved by the Louis Stokes Cleveland Department of Veterans Affairs Medical Center Institutional Review Board, and all subjects gave their written informed consent to participate and for the publication of any potentially identifiable images included in this article.

**Table 1:** Table of subject characteristics.

**Subject**	**Height (m)**	**Weight (kg)**	**Age (year range)**
S1	1.62	56	46–50
S2	1.8	72.7	66–70
S3	1.7	68.2	26–30
S4	1.72	69.5	30–35
S5	1.87	70.5	30–35
S6	1.75	82.5	26–30

### 2.4. Experimental Procedure

Each subject completed two testing sessions. For each testing session, a single condition—friction compensated or uncompensated—was chosen at random and tested over three different speeds, 0.4, 0.8, 1.2 m/s. These three speeds were chosen as follows: 0.4 m/s is the walking speed that other commercial exoskeletons are capable of achieving (Miller et al., 2016). Walking at 0.8–1.2 m/s is considered to be the minimum to be a community ambulator (Robinett and Vondran, 1988; Lapointe et al., 2001).

**Figure 3:**
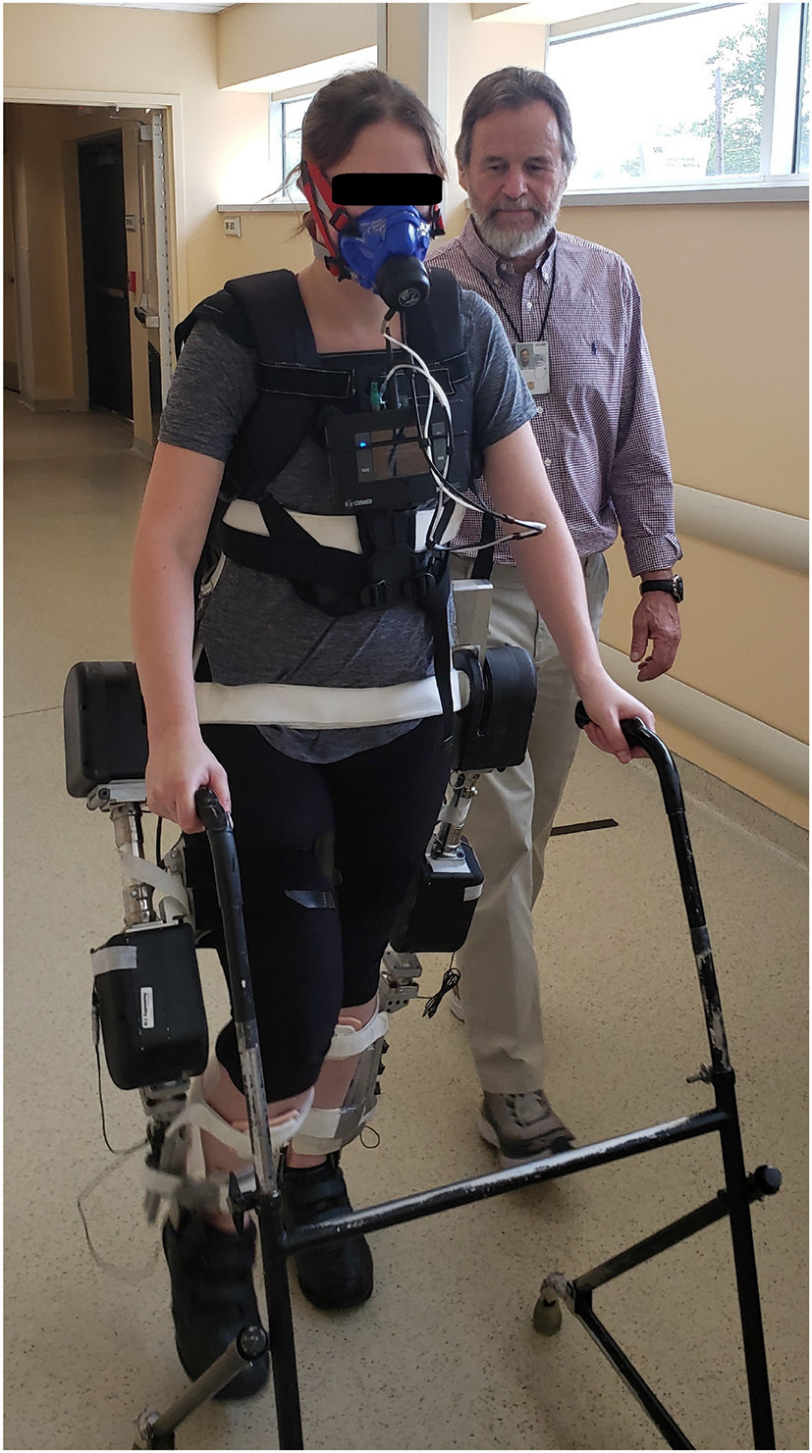
An able-bodied pilot in the MAHNP wearing the K5 Metabolic Analyzer.

Each experiment session began with the subjects donning the K5 metabolic analyzer and breath-mask (COSMED, Rome, Italy). They then donned the MAHNP, placing themselves into the orthotic corset and fastening straps located along the corset, thigh uprights, and ankle-foot orthosis to secure the torso and keep the lower extremities aligned with the system. The ankle-foot orthoses were unlocked to allow free movement at the ankle joints. Adjustments were made to ensure safety, comfort, and proper fit during the experiment. Figure 3 shows the MAHNP donned by an able-bodied pilot.

After donning, subjects sat on a chair and rested for 5 min to acquire a metabolic baseline while wearing the exoskeleton. They then walked on a flat, level hallway at 0.4 m/s, with the chosen experimental condition, uncompensated or friction compensated, being active on the exoskeleton. Participants were not informed of what condition was being tested. The nominal course was 80 m long and based on the 6-min walk test standard (ATS, 2002). Subjects walked around the course for at least 6 min in order to achieve metabolic steady state for at least 2 min. To control for speed over the course of the trial, the floor was marked at 10 m intervals. An experiment administrator kept track of the amount of time and number of steps it took for the subjects to walk between markers, and informed them to adjust their speeds accordingly to match the desired value.

After finishing each walk, subjects sat for 5 min to reacquire the metabolic baseline. This process was repeated while walking at 0.8 and 1.2 m/s, starting at lower speeds to minimize fatigue at the faster conditions. To avoid fatigue and any cumulative metabolic effects that may confound the data, each session only tested a single condition. The alternate exoskeleton condition was tested on a consecutive day, or as close in time to the original session as possible, to minimize potential for inter-condition variation.

In each trial, METS and heart rate were measured by the K5, while the time and step count for each 10 m interval were recorded manually. Step length and cadence were derived from the step counts, number of splits and the split times. Sensors integrated into the exoskeleton joints captured joint kinematics in terms of hip and knee angles and angular velocities at 100 Hz.

### 2.5. Statistical Analysis

An ANOVA was performed to determine differences between metabolic consumption and walking outcomes with and without friction compensation at the tested speeds. Fixed factors included friction compensation (with and without) and walking speed (0.4, 0.8, 1.2 m/s) while subjects was included as a random factor. In addition to individual effects, the model tested for an interaction effect between walking speed and friction compensation. Each subject performed one trial at each condition, and a power analysis (α = 0.05, β = 0.80, effect size = 2 METs), indicated that six subjects were required to demonstrate an effect for the primary outcome measure (metabolic consumption). The same statistical model was implemented with the walking outcome measures to determine potential changes in walking that may contribute to variations in metabolic contributions.

## 3. Results

### 3.1. Metabolic Measures

**Figure 4:**
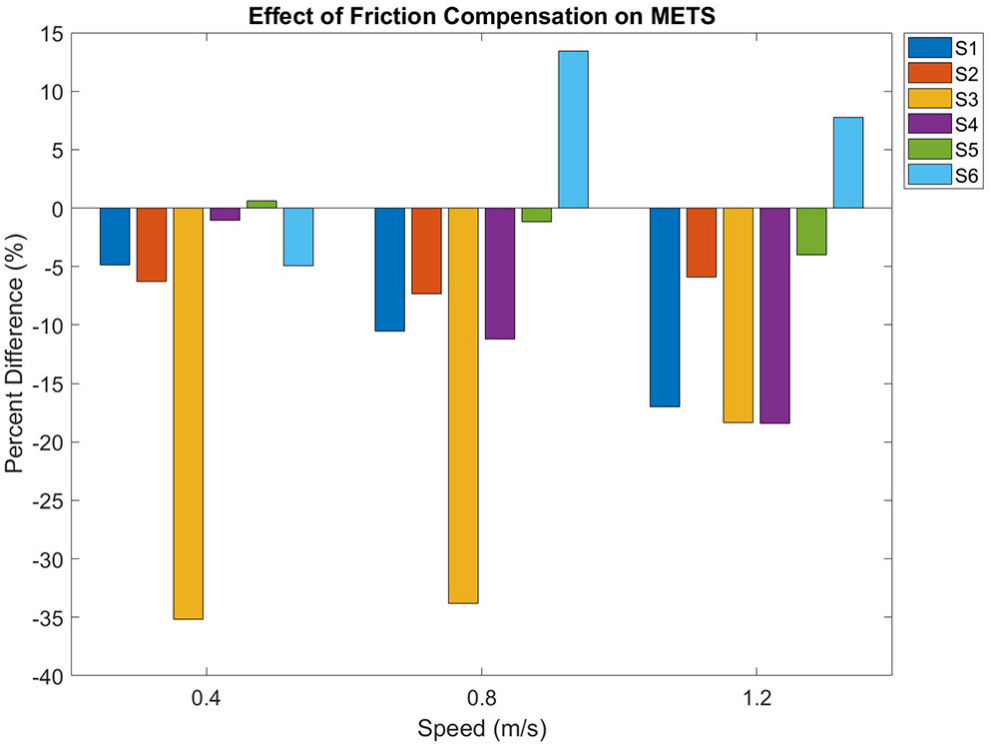
METs percent difference between uncompensated and friction compensated conditions.

Friction compensation reduced metabolic consumption while walking in the exoskeleton (*p* = 0.01). There was an increased consumption at each progressive speed (*p* < 0.01), but there was no interaction effect between friction compensation and walking speed (*p* = 0.7). Figure 4 displays the percent difference in METs between the conditions at all speeds, in terms of the percent difference between the conditions. The percent differences between compensated and uncompensated conditions were calculated using the following equation:

(3) $$\begin{array}{r} p=\left(f-u\right)/u\cdot 100 \end{array}$$

where *p* is the percent difference, *f* is the heart rate or METs under the friction compensation condition at one particular speed, and *u* is the uncompensated value at that same speed. Over all subjects and all gait speeds, there was an average METs decrease of 8.8 ± 12.4 % while walking with friction compensation.

**Figure 5:**
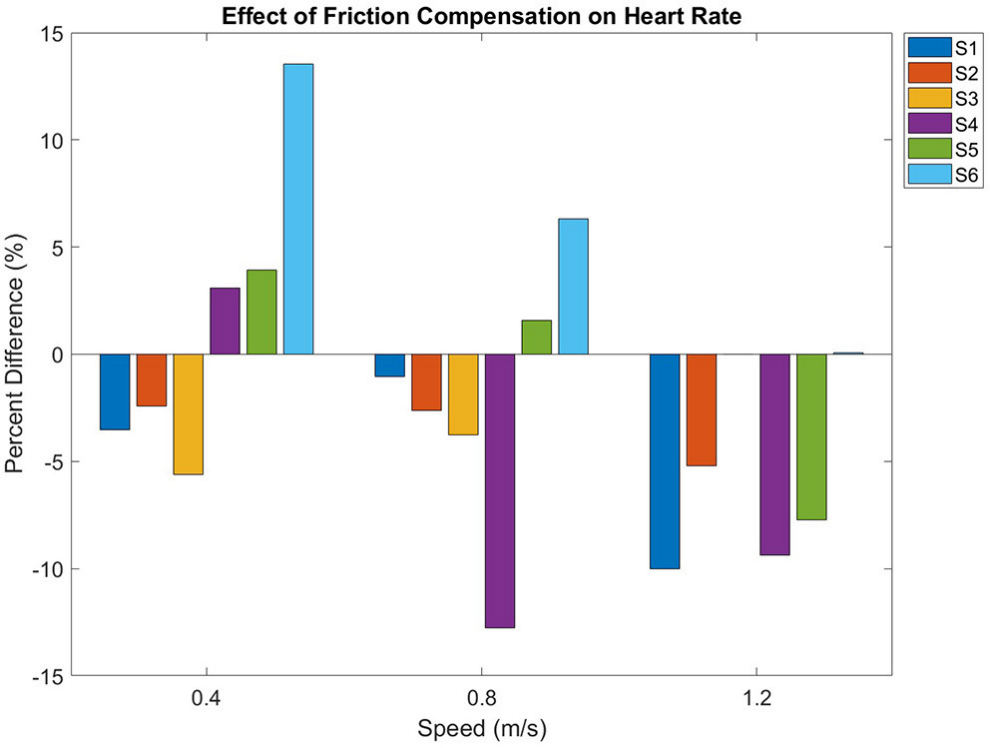
Heart Rate percent difference between uncompensated and friction compensated conditions.

Figure 5 shows the effect of friction compensation on heart rate over all speeds. Heart rate increased with walking speed (*p* < 0.01). However, friction compensation did not have a significant effect (*p* = 0.089) on heart rate. Subject S3's heart rate monitor malfunctioned during walking at 1.2 m/s in the uncompensated condition, so the uncompensated data at this speed were not included in the statistical analysis.

### 3.2. Gait Measures

All subjects were able to attain the target gait speed for each trial condition (Table 2). There was no statistically significant difference between walking speed, cadence, and step length with and without friction compensation (*p* = 0.18, *p* = 0.19, *p* = 0.45). All three gait parameters increased with gait speed (*p* < 0.05).

**Table 2:** Average gait speed, cadence, and normalized step length for each target speed and condition.

	**Actual gait speed (m/s)**		**Cadence (steps/min)**		**Step length (m)**	
**Target gait speed (m/s)**	**Uncomp**	**Friction comp**	**Uncomp**	**Friction comp**	**Uncomp**	**Friction comp**
0.4	0.47 ± 0.03	0.46 ± 0.02	52.4 ± 5.3	51.3 ± 5.9	0.56 ± 0.07	0.55 ± 0.08
0.8	0.78 ± 0.03	0.77 ± 0.04	69.5 ± 4.5	67.5 ± 6.8	0.67 ± 0.05	0.68 ± 0.08
1.2	1.18 ± 0.05	1.16 ± 0.06	94.2 ± 5.3	89.0 ± 7.5	0.76 ± 0.02	0.79 ± 0.04

For the 0.4 m/s condition, the speeds obtained across all subjects were 0.06–0.07 m/s above the target speed. Average speeds recorded were 0.47 ± 0.03 m/s for the uncompensated condition, and 0.46 ± 0.02 m/s under friction compensation. For the 0.8 and 1.2 m/s trials, the mean gait speeds attained across all subjects were slightly less than the target speed, with uncompensated walking at (0.78 ± 0.03 and 1.18 ± 0.05 m/s) and friction compensated walking at (0.77 ± 0.04 and 1.16 ± 0.06 m/s).

Walking cadence was on average 52.4 ± 5.3 steps/min at 0.4 m/s, 69.5 ± 5.4 steps/min at 0.8 m/s, and 94.2 ± 5.3 steps/min at 1.2 m/s. Step length increased with target speed, with step length ranging from 0.56 to 0.76 m in the uncompensated condition, and 0.55 to 0.79 m in the friction compensation condition. Height normalized step length was also not significantly different between friction compensation conditions.

### 3.3. Kinematic Measures

Kinematic improvements during friction compensation are shown in Tables 3, 4. Peak hip angles increased with friction compensation (*p* = 0.014). Peak knee angles were not statistically different based on gait speed (*p* = 0.27). However, peak swing phase knee angle increased by an average of 15.6 ± 7.7 ° during friction compensation (*p* < 0.01). Peak swing phase hip velocity increased with faster gait speeds (*p* < 0.01) and by 34.7 ± 23.1 ° with the addition of friction compensation (*p* < 0.01). Peak swing phase knee flexion velocity increased with gait speed (*p* < 0.01) and by an average of 51.8 ± 59.0 °/s with friction compensation (*p* < 0.01).

**Table 3:** Average peak hip angles and velocities attained by subjects per condition over each gait speed.

	**Hip angle (deg)**		**Hip velocity (deg/s)**	
**Target gait speed (m/s)**	**Uncomp**	**Friction comp**	**Uncomp**	**Friction comp**
0.4	33.6 ± 5.7	36.6 ± 6.0	107.4 ± 20.4	139.6 ± 23.7
0.8	37.4 ± 5.2	41.3 ± 7.0	149.1 ± 26.1	198.6 ± 25.1
1.2	44.5 ± 6.8	46.7 ± 7.4	220.4 ± 30.7	242.7 ± 8.3

**Table 4:** Average peak knee angles and velocities attained by subjects per condition over each gait speed.

	**Knee angle (deg)**		**Knee velocity (deg/s)**	
**Target gait speed (m/s)**	**Uncomp**	**Friction comp**	**Uncomp**	**Friction comp**
0.4	35.2 ± 6.5	52.51 ± 8.8	122.4 ± 19.0	187.4 ± 28.7
0.8	37.40 ± 6.8	54.9 ± 8.0	176.5 ± 28.1	248.0 ± 20.5
1.2	40.6 ± 7.1	52.8 ± 10.5	243.3 ± 42.2	262.3 ± 93.1

## 4. Discussion

The average metabolic consumption across all subjects and speeds decreased by 8.8 % when friction compensation was applied. However, even with the application of friction compensation, walking at 0.4 m/s in the MAHNP required an average of 3.8 ± 0.6 METs across all subjects. This is comparable to walking at 1.3 m/s, which was reported to take 3.45 METs for able-bodied adults (Waters and Mulroy, 1999). At 1.2 m/s the average METs consumed across all subjects walking in the MAHNP was 7.73 ± 0.9. It follows that walking in the MAHNP at speeds >0.4 m/s required more METs than what was utilized by able-bodied individuals walking at speeds required for community ambulation (0.8–1.2m/s) (Robinett and Vondran, 1988; Lapointe et al., 2001). It is likely that metabolic consumption would increase for SCI pilots compared to able-bodied pilots in the MAHNP, as metabolic rate differs between these two populations. This implies that other exoskeleton constraints could contribute to the 4.28 MET difference between typical values of able-bodied walking and walking with the exoskeleton at 1.2 m/s. The exoskeleton enforced a sagittal plane constraint, had added mass at the hip and knee joints and a backpack, and had a corset that prevented pelvic rotation and trunk motions, which could all contribute to increased metabolic cost. These factors could be isolated to determine each factor's effect on metabolic consumption in future work.

When the friction compensation condition was applied to the exoskeleton, kinematic measures were closer to values reported for natural gait. Knee flexion in the uncompensated condition ranged from 35 ° to 40 ° across all subjects, below the 60 ° peak flexion angles reported for able-bodied gait at a natural cadence (Winter, 1987). Peak knee flexion angle values in the friction compensated condition were on average 53.4 ± 1.3 ° across all subjects at all speeds, much closer to that reported for natural gait. Interestingly, the subjects exhibited higher peak hip flexion angles (38.5 ± 5.5 ° uncompensated, 41.5 ± 5.1 ° friction compensated) compared to the 20 ° hip flexion seen in natural gait. To achieve increases in gait speed when cadence is below 120 steps per minute, able-bodied individuals increased stride length and cadence equally (Winter, 1987), which agrees with what we have found on average across the subjects in this study.

Although subjects generally exhibited similar overall trends regarding the effects of friction compensation on metabolic and kinematic parameters and their relationships to walking speed, two subjects (S6 and S3) presented some unique responses. Subject S6 seemed to not exhibit any particular trend across speeds in their trials, while S3 had a diminishing trend toward the higher speeds. Subject S6 had an asymmetrical gait and had extremely high hip flexion compared to all other subjects.

To illustrate, S6 walking at 1.2 m/s in the friction compensated condition exhibited 79.6 ± 9 ° peak flexion for their right knee, and 58.5 ± 5.7 ° for their left knee. Their average hip flexion values under the same condition were also much higher than all other subjects (60.9 ± 6.4 ° right hip, 53.1 ± 1.7 ° left hip). Between 0.4 and 0.8 m/s, S6 increased both step length, 0.70 ± 0.24 to 0.86 ± 0.17 m, and cadence, 0.67 ± 0.18 to 0.92 ± 0.19 steps/s, to achieve the higher speed in the friction compensated condition. However, to achieve 1.2 m/s S6 decreased step length and increased cadence relative to 0.8 m/s values (0.73 ± 0.04 m step length, 1.72 steps/s) opposite of what typically occurs during walking (Winter, 1987).

There was no clear explanatory indication in the data for S3's trend of having the largest decrease in METs at 0.4 m/s and the smallest decrease at 1.2 m/s, opposing the trend seen in S1, S2, and S4. Their movements, cadences, and step lengths fell well within the average compared to other subjects. It may be possible that this subject recruits their muscles slightly differently when they walk compared to other subjects, which would have to be measured via electromyography in future experiments.

Compared to other exoskeletons, METs at 0.4 m/s for the MAHNP is similar to METs reported in a study of the ReWalk exoskeleton in which the SCI pilots consumed 3.2 ± 0.49 METs at 0.22 m/s (Asselin et al., 2015). A study of the Indego Exoskeleton with SCI subjects walking at 0.3 m/s reports that it takes 4.6 METsSCI (Evans et al., 2015), defined as oxygen consumption rate, $\overset{∙}{{\text{VO}}_{2}}$, normalized by 2.7 mL/(kg min), the resting oxygen consumption of persons with SCI (Collins et al., 2010). A clinical review of multiple exoskeletons reported that it took an average of 3.3 METs (95 % CI 2.2–4.4) to walk at an average speed of 0.27 m/s (95 % CI 0.22–0.33) (Miller et al., 2016). In both the ReWalk and Indego studies, the forces required for ambulation were produced entirely with the exoskeleton without FNS. The emphasis of the MAHNP is different than these other exoskeletons in that it minimizes exoskeleton constraints in order to maximize FNS-activated muscular effectiveness, and this goal should not be confused with completely minimizing metabolic cost. Thus, this is not a direct comparison between measures in this study and the others, but rather the commercial exoskeleton studies give a lower bound of oxygen consumption if the SCI pilot was completely assisted and exerted no energy by their lower extremity muscles. It can be assumed that a paraplegic individual using stimulation in the MAHNP would have a higher metabolic consumption at identical gait speeds than these reported values of walking completely assisted, as the FNS-activated muscle would increase their metabolic consumption. Minimization of the intrinsic friction of the device ensures that the increase in metabolic consumption for an SCI pilot is primarily due to the FNS activating the lower limbs to produce useful forces that result in ambulatory motion, and that there will a minimum of force wasted overcoming joint friction.

This study has some limitations and suggests future work. Peak knee angular velocities were 262.3 ± 93.1 °/s, about 60 ° faster than the conditions for deriving the friction compensation model. Future work will incorporate the torque to velocity relationship at these higher speeds in the derivation of the compensation model.

Friction compensation was not found to have a significant effect on heart rate, and this is possibly due to high variability in metabolic steady state heart rate between subjects. The power analysis was performed for the primary outcome measure of metabolic consumption, and a power analysis for heart rate may indicate that more subjects are needed. The small number of participants limits the study's ability to evince general trends that may be exhibited in a larger population, and future work would benefit from recruiting more participants to average out inter-subject variability. The performance of an able-bodied pilot in the MAHNP is not a direct proxy for an SCI pilot, but rather a metabolic lower-bound, as people with SCI have higher metabolic consumption relative to able-bodied individuals (Collins et al., 2010). Future work will perform the same 6 min walk tests at similar speeds with SCI pilots.

Metabolic rate varies within individuals on a day-to-day basis, and when possible the experiments were scheduled on consecutive days. Although this did not always occur for various personal and technical reasons during the course of our study. Also, the walking course was 80 m long, and to achieve 6 min of walking, the subjects had to turn around when they reached the end of the track. Depending on how long it took for the subjects to turn around, this could cause fluctuations in the metabolic steady state. The effect of this was ameliorated by instructing the subjects to turn as fast as they felt safe doing so.

One final aspect that could have affected the oxygen consumption was how well the MAHNP was fit to the pilot. Deviation of the center of rotation of the anatomical joints and the joints of the exoskeleton could produce difficulties during movement, possibly increasing metabolic consumption. Quantifying the relative motion between the anatomical joints and exoskeleton joints could be done with inverse kinematic methods (Torricelli et al., 2018), and the effect of this motion on metabolic consumption would be worthwhile to investigate. For this study, the fit of the exoskeleton was confirmed by the investigators prior to collecting data, but should be assessed by a certified orthotist in future studies.

## 5. Conclusion

The MAHNP was designed with backdrivable actuators that reduced passive resistance at the joints in order to maximize the effectiveness of muscular contractions elicited with FNS. This resulted in an actuator design that had a maximum of 6 N m of passive resistance under normal operation. Actively compensating for this passive resistance reduced it even further, significantly lowering metabolic consumption as well as making gait kinematics more natural. Compensating for the 6 N m of passive resistance at the actuators amounted to a statistically significant 8.8 % decrease in METs across all able-bodied subjects and all speeds. Future work will look toward determining and minimizing other factors that may contribute to the metabolic cost, such as powered compensation of distal weight and investigation into designs that relax the strict sagittal plane constraints.

## Data Availability Statement

The raw data supporting the conclusions of this article will be made available by the authors, without undue reservation.

## Ethics Statement

This study was approved by the Louis Stokes Cleveland Department of Veterans Affairs Medical Center Institutional Review Board, and all subjects gave their written informed consent to participate. Written informed consent was obtained from the individuals for the publication of any potentially identifiable images included in this article.

## Author Contributions

RK, R-DR, and MN contributed to the conception and design of the study. R-DR, MN, NM, and RK performed data collection. MN, NM, and R-DR were responsible for analysis of the collected data. R-DR wrote the first draft of the manuscript. All authors contributed to manuscript revision.

## Conflict of Interest

The authors declare that the research was conducted in the absence of any commercial or financial relationships that could be construed as a potential conflict of interest.
